# Casein Kinase 2 Regulates the Intrinsic Activity of L-Type Calcium Currents in Cardiomyocytes

**DOI:** 10.3390/ijms26136010

**Published:** 2025-06-23

**Authors:** Juan Zhao, Marlena Broszczak, Lucie Parent

**Affiliations:** 1 Centre de Recherche de l’Institut de Cardiologie de Montréal, Université de Montréal, Montréal, QC H1T 1C8, Canada; juan.zhao@icm-mhi.org (J.Z.); marlena.broszczak@icm-mhi.org (M.B.); 2 Département de Pharmacologie et Physiologie, Faculté de Médecine, Université de Montréal, Montréal, QC H3C 3J7, Canada

**Keywords:** neonate rat cardiomyocytes, electrophysiology, phosphorylation, calmodulin inhibitors, calmodulin variants

## Abstract

Heart failure is associated with dysregulation in cellular Ca^2+^ that could involve sarcolemmal L-type Ca^2+^ currents (LTCCs). Building on previous observations showing that recombinant Ca_V_1.2 channels are upregulated by phosphorylated calmodulin (CaM) variants, the cellular mechanism(s) underlying this posttranslational modification was investigated in cultured cardiomyocytes. Whole-cell LTCCs decreased by ≈75% after silencing the gene coding for casein kinase 2 (CK2), a constitutively active kinase in cardiomyocytes, or after its pharmacological inhibition. The overexpression of the dominant negative phosphoresistant single, double T79A/S81A, or triple T79A/S81A/S101A CaM variants resulted in a similar inhibition. In contrast, the overexpression of CaM WT or its double T79D/S81D and triple T79D/S81D/S101D phosphomimetic variants curtailed the downregulation of LTCCs caused by CK2 partial knockdown, suggesting that CK2 is responsible for the posttranslational modification of these CaM target residues. Catecholamines, triggering the protein kinase A (PKA) cascade, partially rescued LTCCs treated with siRNA without or after the overexpression of either CaM WT or stimulating CaM phosphomimetic variants. More importantly, they thwarted the negative impact of the phosphoresistant CaM variants, altogether arguing that CK2 and PKA are acting in synergy to regulate the activity of LTCCs. We conclude that CK2-mediated phosphorylation processes exacerbate the Ca^2+^ load associated with heart failure.

## 1. Introduction

Heart failure is an irreversible condition characterized by the heart’s inability to adequately pump oxygenated blood to meet the body metabolic requirements [1]. It results from a contingent of cardiac pathologies and remains a major burden on the health care system with a high incidence of morbidity and mortality. Approximately one in four persons will develop heart failure in their lifetime, and the prevalence rate is expected to increase among 65- to 70-year-olds [2]. Neurohormonal dysregulation and aberrant calcium ions (Ca^2+^) handling are only two of the several processes involved in its development [3]. Ca^2+^ serves a broad range of functions in cardiac cells over a wide range of time scales. The beat-to-beat rise and fall of intracellular Ca^2+^ in cardiomyocytes govern contraction and relaxation at typical frequencies of once per second. L-type Ca^2+^ channels constitute one of the key Ca^2+^ signaling pathways in cardiac myocytes. They play a crucial role in initiating coordinated cardiac contraction during the systole by handling the influx of Ca^2+^ into cardiomyocytes in response to depolarization during phase 2 of the cardiac action potential [4]. The voltage-gated L-type calcium channel Ca_V_1.2 is expressed in the T-tubules such that localized Ca^2+^ entry triggers a sustained and more global Ca^2+^ release by the sarcoplasmic reticulum in the dyadic cleft [5]. Cardiac L-type Ca_V_1.2 channels are heteromultimeric protein complexes formed by the pore-forming Ca_V_α1C subunit bound to a single extracellular Ca_V_α2δ1 auxiliary subunit [6,7,8]. The pore-forming Ca_V_α1C subunit, encoded by the *CACNA1C* gene, is formed by a single polypeptide chain of 24 transmembrane helices grouped into four structural homologous domains (domains I, II, II, and IV). The cytoplasmic Ca_V_β [9] binds with nanomolar affinity to the “AID” region located in the proximal segment of the first intracellular linker [10,11] that also supports interaction with various intracellular signaling proteins [12,13,14,15,16]. Although not a designated auxiliary subunit, calmodulin (CaM) contributes to Ca^2+^-dependent facilitation and Ca^2+^-dependent inactivation of Ca_V_1.2 [17,18] through binding to the isoleucine–glutamine (IQ) motif in the C-terminal tail of Ca_V_α1C [19,20,21,22]. The potentiating form of CaM-dependent facilitation or upregulation is observed in native cardiac L-type channels during trains of depolarization [23,24] but is usually not reported in recombinant systems with the intact Ca_V_1.2 WT channel [18,25]. Healthy cardiomyocytes achieve Ca^2+^ homeostasis in part by finely adjusting Ca^2+^ influx through this ion channel by modulating the function and/or cell surface expression of any of the ancillary subunits forming the Ca_V_1.2 channel complex [26,27,28,29,30,31].

Beyond the direct interaction of canonical subunits with the pore-forming subunits, channel function can also be indirectly modulated through the posttranslational modification of signaling proteins. Protein kinase-A (PKA) mediates the phosphorylation of Rad, a member of the RGK family, thus conferring the adrenergic receptor agonist-mediated stimulation of LTCCs by relieving the tonic inhibition that unphosphorylated Rad exerts on the intracellular Ca_V_β [32]. In ventricular cardiomyocytes, PKA and calmodulin kinase II (CaMKII) drive the phosphorylation of a host of proteins regulating Ca^2+^ homeostasis and excitation–contraction coupling [33,34,35,36,37].

We have recently reported that overexpressing calmodulin (CaM) or its phosphomimetic T79D and S81D surrogates significantly enhanced the functional activity of recombinant Ca_V_1.2 channels in HEK293T cells through an increase in peak current density and a leftward shift in the activation gating [38]. It is well known that CaM modulates the function of the L-type Cav1.2 (for reviews [39,40]), but our observations were the first to associate the upregulation of CaM on channel function to its phosphorylation status [41,42]. In vitro assays have established that CaM is phosphorylated by the constitutively active Ser/Thr protein kinase CK2, which is alternatively called casein kinase 2 or CKII [43]. CK2 modifies CaM at specific sites, primarily targeting Thr79 and Ser81 within the flexible central linker that connects the N and C lobes of CaM [43].

We have explored the hypothesis that CK2 is actively involved in regulating channel function using cultured rat ventricular cardiomyocytes. Herein, we show that the expression of the CK2 protein is required to support the normal activity of endogenous L-type Ca^2+^ channels. The action of CK2 was emulated by phosphomimetic CaM analogues with the strongest effect resulting from the expression of the triple T79D/S81D/S101D variant. In contrast, preventing the phosphorylation of CaM by overexpressing any dominant negative phosphoresistant CaM variant or silencing the expression of CK2 significantly curtailed the activity of LTCCs by more than 70%. The catecholamines-mediated function of PKA alleviated the negative impact of CK2 suppression and potentiated channel function, even in the presence of the triple phosphomimetic surrogate, supporting the synergy of the two modulation pathways. Our observations further expose the unique and complex role of the ubiquitous CaM protein in regulating the function of LTCCs in cardiomyocytes.

## 2. Results

### 2.1. CaM Increases L-Type Ca^2+^ Current Density in Neonatal Rat Cardiomyocytes

**Figure 1:**
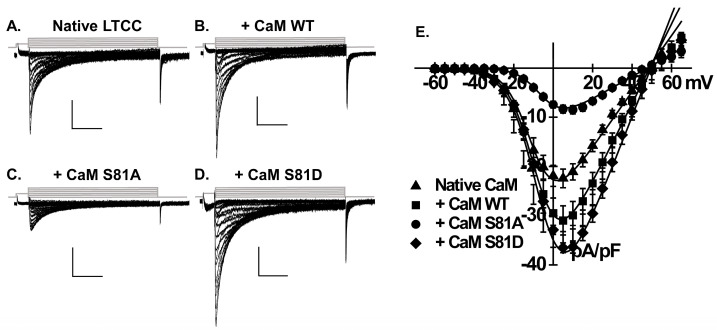
Functional characterization of LTCC currents in neonatal cardiomyocytes overexpressing CaM WT or variants. Representative LTCC current traces are recorded from neonatal rat cardiomyocytes under four conditions: with native/endogenous CaM (**A**), overexpressing CaM WT (**B**), CaM S81A (**C**) or CaM S81D (**D**). Following a 40 ms prepulse to −40 mV to inactivate fast Na^+^ and T-type Ca^2+^ currents, L-type Ca^2+^ currents were elicited by depolarizing voltage pulses ranging from −60 to +65 mV, starting from a holding potential of –80 mV (protocol shown in the inset above the current traces). The time scale is set to 100 ms, and the current density scale is 10 pA/pF throughout. The corresponding peak current densities were plotted against the applied voltages and fitted by a BoltzIV equation (**E**). The current density increased from −22 ± 6 pA/PF in control (native CaM) to −31 ± 6 pA/pF in overexpressing CaM WT (*p* < 0.01). The overexpression of phosphoresistant CaM S81A effectively nullified the upregulation of LTCC currents induced by CaM WT, leading to a significant reduction in peak current density to −9 ± 3 pA/pF (*p* < 0.01). Conversely, the overexpression of phosphomimetic CaM S81D led to a significantly larger LTCC current compared to CaM WT, achieving a peak current density of −37 ± 3 pA/pF (*p* < 0.01).

In a typical recombinant cellular environment, the structural plasticity in which calmodulin (CaM) binds to its targets can vastly surpass the levels of free endogenous CaM [44]. The overexpression of CaM in HEK293T (alternatively referred to as tsA-201) cells [38,45,46,47] or in ventricular cardiomyocytes [48,49] effectively competes with endogenous CaM. It ultimately proved to successfully elucidate the mechanistic actions of CaM on native or recombinant voltage-gated L-type Ca^2+^ channels, namely its pivotal role in the Ca^2+^-dependent inactivation process. In this regard, we have recently demonstrated that overexpressing CaM or its phosphomimetic surrogates in HEK293T cells significantly enhanced the functional activity of recombinant Ca_V_1.2 channels [38]. To investigate the functional relevance of the mechanism, we overexpressed wild-type CaM (CaM WT) as well as CaM Ser81 phosphoresistant and phosphomimetic variants in neonatal rat cardiomyocytes. Figure 1A,B illustrate the typical L-type Ca^2+^ currents recorded from 3-day cultured neonatal rat cardiomyocytes with either native/endogenous CaM (Figure 1A) or after the overexpression of CaM WT (Figure 1B) with 2 mM Ca^2+^ as the charge carrier. The cultured rat cardiomyocytes model has proven to be a robust experimental model for reliably recording endogenous voltage-activated L-type Ca^2+^ currents with typical properties [50] while facilitating liposome-mediated gene transfection and the expression of small plasmids (<4 kb) [51]. As seen, overexpressing CaM WT resulted in a 1.4-fold increase in peak current density, rising from −22 ± 6 pA/pF (endogenous CaM) to −31 ± 6 pA/pF (*p* < 0.01) (Figure 1E and Table 1). Furthermore, the activation gating properties, exemplified by the half-maximal activation voltage (*E_0.5,act_*), remained unaltered despite the overexpression of CaM WT (Table 1).

### 2.2. Phosphorylation of CaM Stimulates LTCCs

Thr79 and Ser81 have been identified as the potential phosphorylated residues within the flexible central linker of CaM. This region is crucial for CaM’s interaction with target CaM-dependent proteins [43,52]. Figure 1C,D are representative LTCC current traces recorded from cells overexpressing the phosphoresistant CaM S81A and phosphomimetic CaM S81D. The overexpression of CaM S81A nullified the upregulation of LTCCs observed with CaM WT, resulting in a current density of −9 ± 3 pA/pF (*p* < 0.01 when compared with CaM WT) and induced a rightward shift of the *E_0.5,act_* to −4 ± 2 mV (*p* < 0.01) (Table 1). A reduction in LTCC current density was observed in cells overexpressing phosphoresistant CaM T79A, which is similar to the effect seen with CaM S81A. Conversely, the overexpression of single phosphomimetic CaM S81D or T79D significantly resulted in an additional 20–25% surge in peak current density (Table 1) compared with CaM WT (*p* < 0.01).

The CaM-induced modulation of LTCCs strongly suggests a distinct role for the phosphorylated form of CaM, as we had previously shown for recombinant Ca_V_1.2 channels [39]. CaM undergoes phosphorylation in vitro and in vivo by multiple protein serine/threonine and protein tyrosine kinases. CK2 is one of the well-established protein serine/threonine kinases involved in this intricate process [52]. The predominant form of CK2 is a heterotetrameric complex, consisting of two catalytic subunits (α and/or α’) and a dimer of regulatory CK2β subunits [53]. CK2 is known to phosphorylate a substantial fraction of endogenous CaM in vivo with phosphorylation levels ranging from 10% to 45% [54,55]. To elucidate the role of CK2-mediated calmodulin phosphorylation in modulating the function of LTCCs, we conducted experiments utilizing 4,5,6,7-tetrabromobenzotriazole (TBB), which is a cell-permeable highly selective ATP/GTP-competitive CK2 inhibitor [56]. We reasoned that if CK2 is involved in the phosphorylation of CaM, TBB could prevent the CaM-induced increase in peak current density.

**Figure 2:**
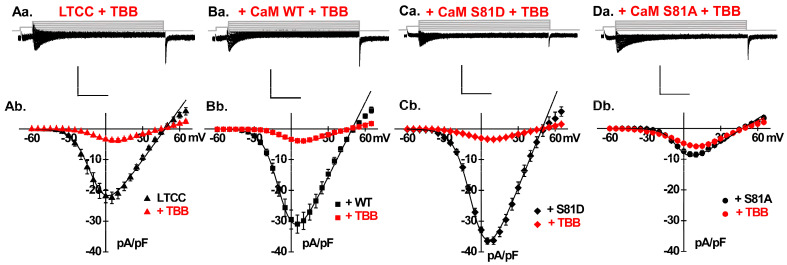
Impact of selective CK2 inhibitor TBB on LTCC currents in neonatal cardiomyocytes. Representative LTCC current traces were recorded from neonatal cardiomyocytes treated with 0.5 µM TBB under 4 conditions: with native/endogenous CaM (**Aa**), overexpressing CaM WT (**Ba**), CaM S81D (**Ca**) or CaM S81A (**Da**). In cells expressing native/endogenous CaM, TBB treatment induced a significant 80% reduction in peak current density, from −22 ± 6 pA/pF in control to −4 ± 2 pA/pF post-treatment (*p* < 0.01), as shown in (**Ab**). Similarly, TBB treatment significantly reduced the enhanced LTCC currents induced by overexpressing CaM WT (as shown in Figure 1B), resulting in an approximately 90% reduction in peak current density, from −31 ± 6 pA/pF under control conditions to −3.9 ± 1 pA/pF post-treatment (*p* < 0.01), as depicted in (**Bb**). Likewise, the LTCC current significantly increased by overexpressing the phosphomimetic CaM S81D (as shown in Figure 1D), which was markedly reduced following TBB treatment, achieving a peak current density of −3 ± 1 pA/pF (*p* < 0.01), as depicted in (**Cb**). For cells overexpressing the phosphoresistant CaM S81A (as shown in Figure 1C), TBB treatment further decreased the already nullified LTCC current, resulting in a current density of −6 ± 1 pA/pF post-treatment (*p* < 0.01), as shown in (**Db**).

Figure 2Aa depicts representative LTCC whole-cell current traces recorded from neonatal rat cardiomyocytes with native CaM, following a 15-minute incubation with 0.5 µM TBB. TBB caused a significant reduction in peak current density of approximately 80%, decreasing from −22 ± 6 pA/pF to −4 ± 2 pA/pF (*p* < 0.01), and induced a rightward shift in the activation gating of LTCCs from −10 ± 3 mV to −5 ± 2 mV (*p* < 0.01) (Table 1) (Figure 2Ab). TBB also markedly attenuated the upregulation of LTCCs in cells overexpressing CaM WT (Figure 2Ba,Bb), achieving an 87% reduction in current density from −31 ± 6 pA/pF to −4 ± 1 pA/pF (*p* < 0.01) (Table 1). Additionally, TBB elicited a rightward shift in the activation gating from −8 ± 3 mV to −3 ± 2 mV (*p* < 0.01), rendering L-type currents slightly more resistant to open within a physiological range of membrane potentials.

### 2.3. Silencing CK2 Expression Prevents CaM-Induced Functional Modulation of LTCCs

CK2 phosphorylates CaM at specific sites in vitro, preferentially targeting in vivo Ser81, which is followed by Thr79 and Ser101 in that order [54]. We explored the impact of TBB on LTCCs in cells overexpressing CaM phosphomimetic or phosphoresistant variant. As seen, TBB application also led to a dramatic reduction of over 90% in the LTCC peak current density in cells overexpressing CaM S81D (*p* < 0.01) (Figure 2Ca,Cb). In contrast, upon the overexpression of CaM S81A, the reduction in LTCC current density was significantly lessened to just 33% with values decreasing from −9 ± 3 pA/pF to −6 ± 0.8 pA/pF (*p* < 0.01) (Figure 2Da,Db). Overall, the 80–90% inhibition of LTCCs in cells with endogenous CaM, overexpressing CaM WT, or phosphomimetic CaM T79D or S81D contrasted with the significantly lower 33% inhibition in cells overexpressing phosphoresistant CaM T79A or S81A (Table 1).

**Figure 3:**
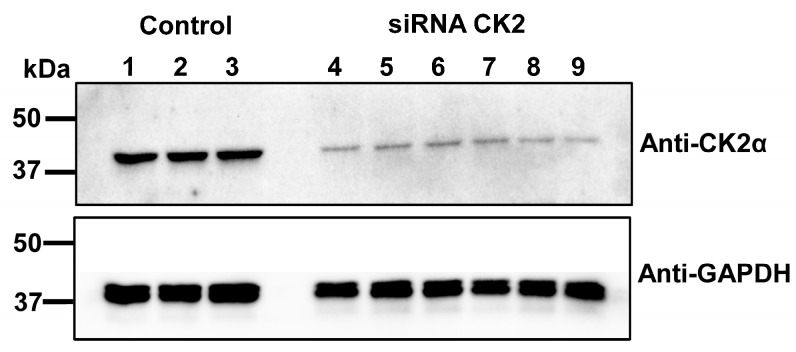
Western blot analysis of cardiomyocyte protein samples. Lanes 1–3 contain control cardiomyocyte lysates, while lanes 4–9 contain total protein lysates from cardiomyocytes transfected with siRNA directed toward CK2. These wells contain proteins from transfected cardiomyocytes sourced from 5 different culture preparations. The transfections and protein extraction procedures were performed over the course of several months to ensure consistency and reproducibility. The membrane was incubated with primary Anti-Casein Kinase 2α (1: 1000) and secondary Anti-Rabbit (1: 10,000) antibodies; after, it was stripped and reprobed with Anti-GAPDH (1: 10,000) and secondary Anti-Rabbit (1: 10,000) antibodies to confirm protein loading. Protein bands were detected using enhanced chemiluminescence (ECL) and visualized by the ChemiDoc Imaging System (Bio-Rad, Hercules, CA, USA). Cardiomyocytes exposed to the siRNA showed a 89.6 ± 3.2% (mean ± SD) decrease in the band intensity of the CK2 protein when normalized to the expression of the housekeeping protein GAPDH.

Given that TBB also exhibits off-target effects on other protein kinases, such as phosphorylase kinase, glycogen synthase kinase 3β, cyclin-dependent kinase 2/cyclin A, and PKA, albeit with a moderate to low potency range [56], we employed siRNA to silence the Csnk2α1 gene. This approach targets the knockdown of CK2 expression to isolate its specific effects on LTCCs in neonatal cardiomyocytes. For these experiments, 10 nM siRNA targeting the casein kinase 2 α1 (*Csnk2a1*) gene (Ambion, siRNA ID # s138044) was transfected in freshly isolated cardiomyocytes with or without cDNA coding for CaM WT or variants. The procedure significantly attenuated by near 90% the total protein expression of endogenous CK2, which was validated by Western blotting the total cardiomyocyte lysates collected 65–75 h post-transfection (Figure 3). 

**Figure 4:**
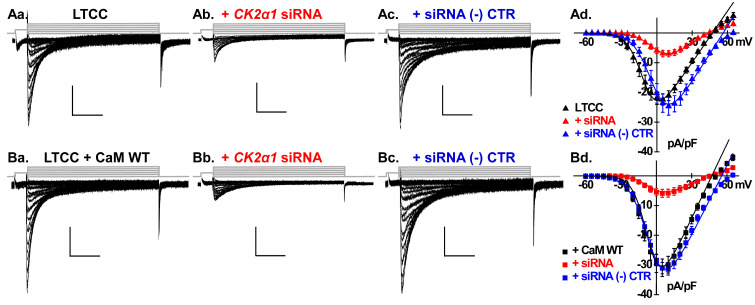
Functional characterization of LTCC currents in neonatal cardiomyocytes post-*Csnk2α1* gene silencing elucidated the pivotal role of CK2 in phosphorylating both native and heterologous expressed CaM. (**Aa**) Representative LTCC current trace recorded from cells expressing native/endogenous CaM. (**Ab**,**Ac**) Representative LTCC current traces from cells transfected with either the specific *Csnk2α1* siRNA or universal negative control siRNA. In cells where the *Csnk2α1* gene was silenced, the LTCC current density significantly decreased to −7 ± 3 pA/pF (*p* < 0.01), in contrast to cells transfected with the universal negative control siRNA, which showed no significant change. (**Ba**–**Bc**) LTCC current traces from cells overexpressing CaM WT under the same experimental conditions as those in (**Aa**–**Ac**). Subsequent to *Csnk2α1* gene silencing, the LTCC current density decreased to only 20% of the control level, with a value of −6 ± 2 pA/pF (*p* < 0.01), whereas no significant change in current density was observed in cells transfected with the universal negative control siRNA. The corresponding current densities were plotted against the applied voltages and fitted using the BoltzIV equation as shown in (**Ad**,**Bd**).

**Figure 5:**
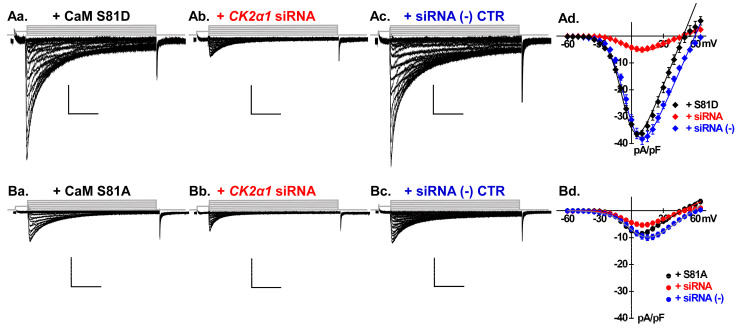
Functional characterization of LTCC currents post-*Csnk2α1* gene silencing in neonatal cardiomyocytes overexpressing CaM variants S81A or S81D. (**Aa**–**Ad**) LTCC current traces from cells overexpressing CaM S81A under three distinct conditions. (**Aa**) is the baseline CaM 81A current, while (**Ab**,**Ac**) are CaM S81A currents from cells co-transfected with the *Csnk2α1* siRNA or the universal negative control siRNA, respectively. The corresponding current–voltage are displayed in (**Ad**). Silencing the *Csnk2α1* gene led to a significant reduction in the CaM S81A current density from −9 ± 3 pA/pF in the control to −5 ± 2 pA/pF (*p* < 0.01). Conversely, cells transfected with the universal negative control siRNA showed no significant change in current density. (**Ba**–**Bd**) illustrate LTCC current traces recorded from cells overexpressing CaM S81D under the same conditions described for (**Aa**–**Ac**). In the *Csnk2α1* gene-silenced cells, the CaM S81D current density dropped to just 15% of the control level, reaching −5 ± 2 pA/pF (*p* < 0.01). In contrast, cells transfected with the universal negative control siRNA exhibited no notable change in current density.

The treatment significantly reduced the native LTCC peak current density by 68% from −22 ± 6 to −7 ± 3 pA/pF (*p* < 0.01) (Figure 4Aa,Ab). *Csnk2a1* knockdown reduced also CaM-boosted LTCCs from −31 ± 6 pA/pF to −6 ± 2 pA/pF (*p* < 0.01) (Figure 4Ba,Bb). In cells overexpressing either CaM T79D or S81D, the reduction was similar, limiting LTCCs to ≈−5 pA/pF (*p* < 0.01) and thus resulting in channel activity comparable for the four single phosphomimetic and phosphoresistant variants (Figure 5Aa–Ad,Ba–Bd, Table 1). Altogether, silencing *Csnk2a1* annihilated the stimulating impact of CaM with an outcome similar with the one observed with the pharmacological agent TBB.

Overexpressing the universal negative control siRNA in neonatal cardiomyocytes did not cause any significant impact on the LTCC current densities recorded with native CaM (Figure 4Ac), with overexpressed CaM WT (Figure 4Bc), CaM S81D (Figure 5Ac), or CaM S81A (Figure 5Bc). It can be seen on the superimposed I/V curves (Figure 4 and Figure 5, panels Ad and Bd) that the universal negative control siRNA caused a rightward shift of approximately 10 mV in the reversal potential (Table 1) that could not be further investigated given the proprietary nature of the control siRNA. Nonetheless, this result confirms that the reductions observed with the siRNA directed against Csnk2α1 were caused by the specific reduction in the CK2α1 expression and were not an artifact of the siRNA delivery process.

### 2.4. LTCCs Are Regulated by the CK2-Mediated Simultaneous Phosphorylation of CaM Thr79 and Ser81

**Figure 6:**
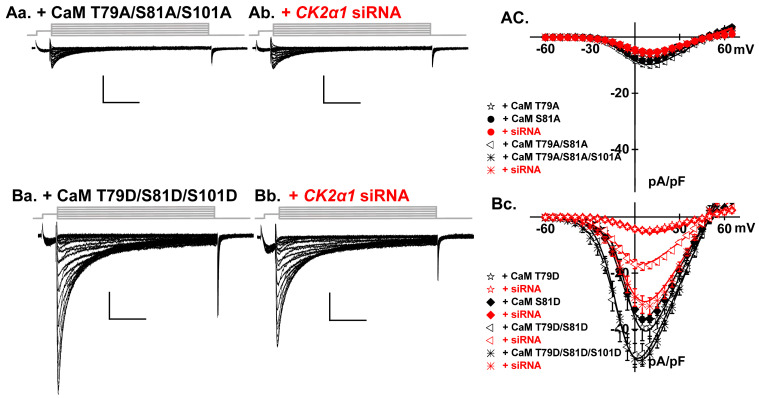
CK2 phosphorylates CaM at multiple target sites. (**Aa**,**Ab**) Representative LTCC current traces from cells overexpressing the phosphoresistant CaM T79A/S81A/S101A with or without *Csnk2α1* gene silencing. Overexpression of CaM T79A/S81A/S101A resulted in a significant nullification of the LTCC currents, leading to a dramatic reduction in peak current density to −6 ± 2 pA/pF. This reduction is notable when compared to cells expressing native CaM (*p* < 0.01) or cells overexpressing CaM WT (*p* < 0.01), as depicted in Figure 1A,B. (**AC**). Knocking out the *Csnk2α1* gene did not induced any further reduction in the LTCC current, when compared to cells overexpressing single phosphoresistant CaM T79A or S81A, maintaining a current density of −6 ± 2 pA/pF. (**Ba**,**Bb**) LTCC current traces from cells overexpressing phosphomimetic CaM T79D/S81D/S101D with or without *Csnk2α1* gene silencing. (**Bc**) The overexpression of phosphomimetic CaM T79D/S81D/S101D led to significantly larger LTCCs compared with the results obtained with native CaM or after transfection with CaM WT, T79D, or S81D, achieving a peak current density of −51 ± 5 pA/pF (*p* < 0.01). *Csnk2α1* silencing failed to obliterate LTCCs under this condition, leaving a 60% residual current density of −32 ± 8 pA/pF (*p* < 0.01) indicating that the simultaneous phosphorylation of these 3 sites could partially reverse the impact of preventing CK2-mediated phosphorylation of LTCCs.

Phosphomimetic CaM variants are considered as constitutively phosphorylated, yet their overexpression did not prevent the inhibition of LTCCs by TBB (Figure 2Ca,Cb) or siRNA technology (Figure 5Aa,Ab). We investigated the additive impact of CaM Thr79 and Ser81 with the double CaM T79D/S81D and T79A/S81A variants. CaM T79A/S81A reduced the LTCC peak current density to the same extent as either single one. In contrast, CaM T79D/S81D significantly surpassed by 40% the impact of overexpressing either CaM T79D or S81D with peak current densities averaging −50 pA/pF (*p*< 0.01) (Figure 6, Table 1).

The role of the consensus phosphorylation site Ser101, located within the EF-hand motif 3 on the C-lobe was explored with triple phosphoresistant CaM T79A/S81A/S101A (Figure 6Aa,Ab) and phosphomimetic CaM T79D/S81D/S101D variants (Figure 6Ba,Bb). CaM T79D/S81D/S101D significantly surpassed the impact of single variants but remained in the same range as the double T79D/S81D (*p* > 0.1, Table 1). Attenuating the expression of the Csnk2α1 gene reduced the peak current density by only 35%, maintaining the same activity as measured by overexpressing CaM WT. CaM T79A/S81A/S101A reduced the LTCC peak current density to −6 ± 2 pA/pF and was not further decreased by the siRNA treatment. CK2 thus targets simultaneously at least two sites.

### 2.5. Catecholamines Partially Rescue CK2 Attenuation

**Figure 7:**
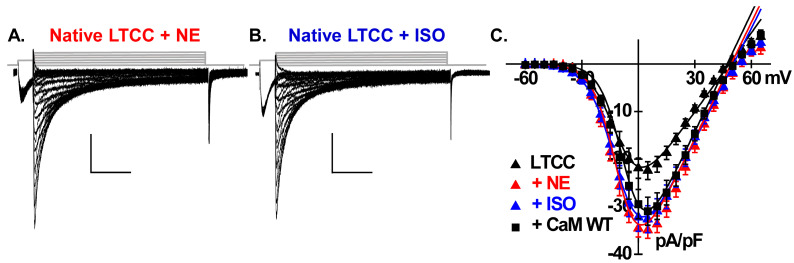
Catecholamines (NE and ISO) promote the activity of LTCCs. (**A**,**B**) Representative LTCC current traces recorded from neonatal rat cardiomyocytes following treatment with 1 µM NE or 100 nM ISO. (**C**) The current–voltage relationships under these conditions with control (native CaM) and overexpressing CaM WT included for comparison. NE treatment significantly increased the current density from −22 ± 6 pA/pF (control) to −35 ± 5 pA/pF (*p* < 0.01). Similarly, ISO treatment resulted in a current density increase to −33 ± 3 pA/pF (*p* < 0.01).

**Figure 8:**
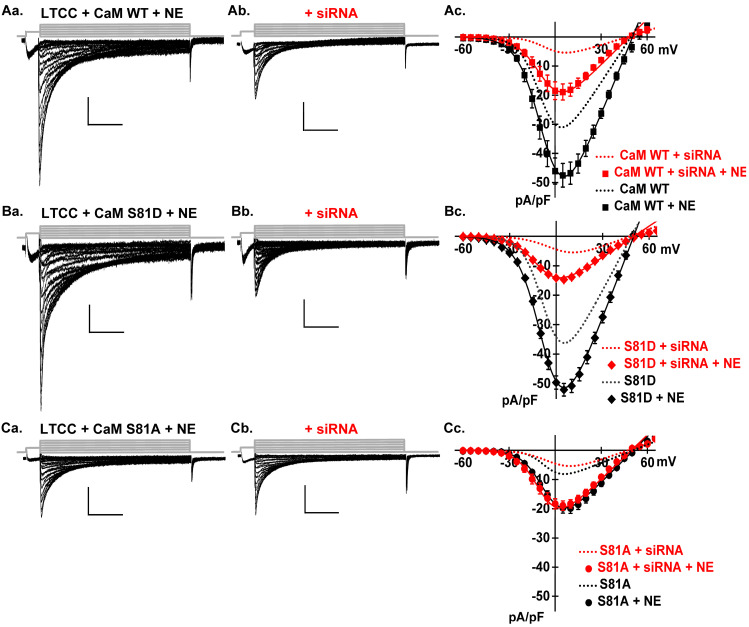
Knocking out the *Csnk2α1* gene does not abrogate the β-adrenergic stimulation of LTCC activity. (**Aa**). The augmentation of LTCC currents by overexpressing CaM WT, as demonstrated in Figure 1B, was potentiated by NE treatment, leading to a peak current density of −48 ± 8 pA/pF (*p* < 0.01). (**Ab**) indicates that *Csnk2α1* gene silencing only partially inhibited the LTCC currents post-NE treatment, leaving a residual current density of −19 ± 4 pA/pF, although it remains significantly reduced compared to CaM WT (*p* < 0.01). The corresponding current–voltage relationships are detailed in (**Ac**), which also includes the I–V curves from cells overexpressing CaM WT alone or subjected to *Csnk2α1* gene silencing for comparison. (**Ba**,**Bb**) LTCC current traces from cells overexpressing CaM S81D, with or without *Csnk2α1* gene silencing, subjected to NE treatment. The upregulation of LTCC currents by overexpressing CaM S81D, as shown in Figure 1D, was further amplified by NE treatment, leading to a peak current density of −52 ± 8 pA/pF (*p* < 0.01). *Csnk2α1* gene silencing partially inhibited these currents, resulting a residual current density of −15 ± 2 pA/pF (*p* < 0.01), which is significantly lower than in CaM S81D. The corresponding current–voltage relationships are detailed in (**Bc**). The I–V curves from cells overexpressing CaM S81D alone or subjected to *Csnk2α1* gene silencing are also included for comparison (dotted lines). (**Ca**,**Cb**). LTCC current traces from cells overexpressing CaM S81A, with or without *Csnk2α1* gene silencing, subjected to NE treatment. Overexpressing CaM S81A nullified LTCC current upregulation, as shown in Figure 1C. NE treatment significantly boosted this to a peak current density of −20 ± 5 pA/pF (*p* < 0.01). *Csnk2α1* silencing did not significantly affect this enhancement, maintaining a density of −19 ± 5 pA/pF. The corresponding current–voltage relationships are detailed in (**Cc**). The I–V curves from cells overexpressing CaM S81A alone or subjected to *Csnk2α1* gene silencing are also included for comparison (dotted lines).

LTCCs are upregulated by β-adrenergic stimulation by increasing the mean open time and/or the open probability as reported at the level of the single channel [57,58]. At the whole-cell level, β-adrenergic receptor stimulation increases LTCC amplitude, shifts the voltage dependence of activation to more negative potentials, and accelerates inactivation rates [49]. Catecholamines bind to β-adrenergic receptors, leading to elevated cyclic adenosine monophosphate (cAMP) levels via the activation of adenylate cyclase, and consequently activation of the cAMP-dependent protein kinase A (PKA) [58]. Recent findings by Papa et al. have identified the small RGK G-protein Rad, an inhibitor of voltage-activated Ca^2+^ channels, as a key target of PKA [14,29,31]. Upon exposure to a β-adrenergic agonist, PKA phosphorylates Rad, leading to its dissociation from the Ca_V_β subunit, thereby relieving its inhibitory effect and enhancing the activity of Ca_V_1.2 [30]. At the structural level, the impact of Rad and CaM converges the intracellular I–II linker of the pore-forming Ca_V_α1C subunit of Ca_V_1.2 [15,38]. Figure 7 shows representative LTCC current traces following exposure to 1 µM norepinephrine (NE) or 100 nM isoproterenol (ISO). LTCCs in NE-treated cells and ISO-treated cells were very similar to −35 ± 5 pA/pF and −33 ± 8 pA/pF peak current densities, respectively, as well as remarkably similar to the mean current densities of −31± 6 pA/pF recorded after the overexpression of CaM WT. All three conditions significantly differed (*p* < 0.01) from the level of −22 ± 6 pA/pF recorded for untreated LTCCs. A minor positive shift in the activation gating properties of LTCCs, as deduced from the *E_0.5,act_* values, was observed in the ISO-treated cells (Table 1). β-adrenergic stimulation was also evaluated in cardiomyocytes overexpressing CaM WT (Figure 8A).

As seen, CaM-upregulated LTCCs were significantly enhanced by 1 µM NE, reaching −48 ± 8 pA/pF (*p* < 0.01) (Figure 8A, Table 1), almost doubling the peak current density. The fraction of the increased activity recorded from NE- and ISO-treated cardiomyocytes resisted the exposure to TBB or siRNA under four experimental conditions (native CaM and overexpression of CaM WT, CaM S81D, or CaM S81A). These findings argue that CK2 and PKA work synergistically through non-overlapping signaling pathways to enhance the activity of LTCCs.

## 3. Discussion

**Phosphorylation of CaM controls the activity of LTCCs in cardiomyocytes**

Calmodulin is the quintessential Ca^2+^ sensor with a near-ubiquitous distribution. It mostly achieves its role through a specific conformational change induced by the high-affinity Ca^2+^ binding onto the four EF-hand motifs. Its rich interactome results in pleiotropic outcomes that are still being deciphered despite the fact that CaM has been studied for more than 40 years [59]. The Ca^2+^ binding properties of CaM have been shown to be essential in optimally regulating total Ca^2+^ influx by controlling the rate of inactivation and promoting the facilitation of LTCCs in ventricular cardiomyocytes under physiological conditions [17,39]. Ca^2+^-bound CaM is also required to activate the Ca^2+^/calmodulin-dependent protein kinase II (CaM-kinase II) [60] that plays a role in the development of heart failure [61,62].

The two-lobe CaM protein hosting the hydrophilic pockets tailored for Ca^2+^ sensing are bound by a flexible central linker featuring two active phosphorylation sites Thr79 and Ser81. Little is known about the functional impact of CaM phosphorylation on cardiac ion channels, besides its well-documented role in the downregulation of SK channels [63]. In vitro assays, performed in the absence of other proteins, demonstrated that the phosphorylation of CaM on serine/threonine decreases its intrinsic ability to activate CaM-kinase II [41]. We have, however, previously shown that phosphomimetic CaM variants stimulate recombinant L-type Ca_V_1.2 channels expressed in HEK293T cells [38]. Herein, we are demonstrating a strong correlation observed at steady state between the native expression of CK2, the phosphorylation status of CaM, and the function of LTCCs in cardiomyocytes.

The phosphorylation of CaM by CK2 is inferred by the functional effect of the CaM variants and in particular by the observation that inhibiting the enzymatic function of CK2 through a pharmacological or a gene silencing strategy yielded the same impact on the function of LTCCs than phosphoresistant CaM variants. Measuring the impact of CK2 on LTCC whole-cell currents in live cells requires to preserve the cell viability and as such circumvents the array of biochemical tools to be employed. The challenge of working with a large membrane protein not easily purified further limits the quantitative estimation of kinetic parameters and the dynamic response of the LTCC function to repeated cycles of the phosphorylation and dephosphorylation of CaM.

Within the relative physiological context, attenuating the expression of CK2 significantly reduced the activity of native LTCCs measured with endogenous CaM and prevented the upregulation of LTCCs observed after the overexpression of CaM WT. The CK2-mediated modulation requires that the flexible loop linking the N- and C-lobes of CaM includes at least one of the three phosphorylation-ready threonine and serine residues at positions 79, 81 and 101. Preventing phosphorylation at one of these sites with a single alanine-substituted CaM variant was sufficient to nearly annihilate channel function without additional impact from a second or third site. In contrast, the overexpression of the phosphomimetic CaM T79D/S81D/S101D analogue nearly doubled the effect of CaM WT on LTCCs and significantly countered but not completely reversed (see below) the impact of silencing the *Csnk2α1* gene. At this time, it remains to be seen whether the phosphorylation of CaM modifies channel function by directly altering its interaction with the pore-forming Ca_V_α1C subunit or indirectly through other partners, for instance membrane-generated PIP2 lipids, as it was shown for small Ca^2+^-activated K^+^ SK channel complexes [64]. Whether directly or indirectly, the role of phosphorylated CaM in promoting cell surface targeting and/or stability of the Ca_V_1.2 complex at the cell surface should be evaluated. Proteomics will be essential to address this issue and goes beyond the scope of this current work. Provided that a direct interaction is demonstrated, the nature of the interaction site and the affinity of this interaction would require additional work. While the structural properties of the IQ domain in the C-terminal, crucial for the Ca^2+^-CaM dependent inactivation process, have been documented [65], CaM is known to bind in vitro to additional sites on the pore-forming α1C of Ca_V_1.2 with lower affinity [40]. We have previously shown that stabilizing the α-helical conformation of the I–II linker reduces the impact of CaM phosphorylation on channel function [38], suggesting that this site plays an active role in the CaM-mediated regulation of LTCCs.

The structural properties of the CaM linker have been reported in the 3CLN.pdb high-resolution X-ray structure of CaM. The presence of a salt bridge linking the side chains of CaM Lys75 and Glu84 delineating the flexible linker can be speculated. This internal salt bridge would create a sharp reverse turn stabilized by a hydrogen bond between the main chain carbonyl oxygen of Asp78 and the peptide nitrogen of Ser81 [66]. The phosphorylation of CaM by virtue of adding a phosphate group could alter the stability of the interaction and modifies its conformation and potentially its interaction with target protein (s).

Of note, the strong correlation between the impact of overexpressing CaM WT and its phosphorylated variants indicates that CaM exists predominantly in its phosphorylated state under physiological conditions. Purified CaM, added at 2 μM, has been previously shown to increase LTCC activity up to 200–250% recorded from inside-out patches isolated from guinea-pigs ventricular cardiomyocytes [67]. CaM prevented the current run-down of LTCCs, and this effect was independent of CaMKII but required intracellular ATP [67]. Altogether, these observations support a mechanism whereby LTCCs are controlled by physiological concentrations of CaM [68] and/or the ratio of phosphorylated versus non-phosphorylated CaM species.

**CK2 and PKA regulate conjointly L-type Ca^2+^ currents in cardiomyocytes**

CK2 was one of the first identified protein kinases (reviewed in [69,70]). Being constitutively active, CK2 does not respond to external stimuli, but it plays a role in mediating the pathological growth of cardiomyocytes, and its modulation of tumor suppressor proteins is controlled by the hypertrophic cascade [71]. CK2 is also activated by ischemia in the preconditioned rabbit heart [72]. Herein, we demonstrate that silencing CK2 reduced LTCCs by 35% despite the “constitutive” phosphorylation of the two to three sites indicating that CK2 is acting either on yet to be identified sites within CaM or acting on other modulatory proteins required for the tonic activity of LTCCs in cardiomyocytes.

Partial inactivation of the CK2 gene or invalidation of any CaM phosphorylation site did not prevent LTCC stimulation by catecholamines, indicating that the two signaling pathways are acting in parallel. It ensures that limiting the CK2-mediated phosphorylation of CaM counteracts the surge in activity prompted by sympathetic stimulation. CK2 and PKA thus support complementary roles, the former setting the basal tone and the latter adjusting the LTCC channel activation in face of a fight or flight situation.

**Future Directions**

CK2 can regulate normal and tumor cell proliferation, and its role in controlling cancer is currently investigated. CK2 inhibitors can also be used to treat neurological and psychiatric disorders. To this list, we suggest that the role of CK2 in the development of heart failure needs to be seriously explored.

## 4. Materials and Methods

**Recombinant DNA Techniques**

The human calmodulin (CaM) (GenBankTM accession number M27319), subcloned in pcDNA3.1 (Thermofisher) vector with consecutive Histidine (His-His-His-His-His-His) and cMyc (Glu-Gln-Lys-Leu-Iso-Ser-Glu-Glu-Asp-Leu) tags in C-terminal, was a gift from Dr Rémy Sauvé, Université de Montréal. The cDNA mutations of CaM were introduced in this vector. CaM is numbered as reported in [64] to account for the absence of the N-terminal Met residue in the mature protein. All cDNA mutations in CaM were produced with the Q5 Site-Directed Mutagenesis Kit (catalog #E0554S, New England Biolabs Inc., Whitby, ON, Canada) according to the manufacturer’s instructions. Briefly, substitutions of nucleotides were created by incorporating the desired mutation in the center of the forward primer, and the reverse primer is designed so that the 5′ ends of the two primers anneal back-to-back. Following the PCR, the amplified DNA is circularized and the template is removed with a kinase-ligase-DpnI enzyme mixture before transformation into high-efficiency NEB DH5-α competent E. coli. All constructs were verified by automated double-stranded sequence analysis (“Centre d’expertise et de services Génome Québec”, 3175 Chemin de la Côte-Sainte-Catherine, Montréal, QC, Canada). The protein expression at the expected molecular weight was confirmed by standard Western blot analysis for each construct after recombinant expression.

**Isolation and primary culture of neonatal rat cardiomyocytes**

The use and care of laboratory rats were conducted according to the Canadian Council for Animal Care and approved by the Animal Care Committee of the Montreal Heart Institute. Immediately after euthanasia, hearts were removed from 1- to 2-day-old Sprague–Dawley rats, and ventricles were minced in dissociation buffer (in mM): 116 NaCl, 20 Hepes, 0.8 Na_2_HPO_4_, 5.6 glucose, 5.4 KCl, 0.8 MgSO_4_, pH 7.35. The tissue was then digested with 0.6 mg/mL pancreatin (Sigma Aldrich, Oakville, ON, Canada) and 0.4 mg/mL Collagenase Type II (Worthington Biochemical Corp, Lakewood, NJ, USA) for 5 min at 37 °C under constant agitation. Thereafter, the supernatant was discarded and replaced with fresh digestion solution (20 min agitation at 37 °C). The supernatant was collected and centrifuged at 1200 rpm for 5 min. The obtained pellet of cells was resuspended in 2 mL horse serum. This digestion step was repeated five times. The collected cells from each digestion were combined, resuspended in culture medium, and passed through a 70 µm nylon filter. The culture medium used was high-glucose Dulbecco’s Modified Eagle Medium (DMEM) (Invitrogen, Burlington, ON, Canada) supplemented with 15% Fetal Bovine Serum and 1% penicillin–streptomycin. The cells were then pre-plated for 75 min to allow fibroblast adhesion in 5% CO_2_ incubator at 37 °C. The cardiomyocyte-enriched suspension (nonadherent cells) was transferred and seeded onto culture plates.

**Cardiomyocyte transfection and treatment**

Neonatal cardiomyocyte transfection was performed at the time of plating. The heterologous expression of CaM WT or variants was achieved by transiently transfecting cDNA mix with 10 µL Lipofectamine 2000 (catalog #11668019, Invitrogen, Carlsbad, CA, USA) in 35 mm Petri dishes containing glass cover slips for optimal transfection efficiency. The volume of lipofectamine had, however, to be reduced from 10 µL to 7 µL when overexpressing the triple phosphomimetic T79D/S81D/S101D CaM variant to improve cardiomyocyte survival, which was presumably to limit large inward Ca^2+^ influxes harmful to the cells. The cDNA mix comprised 2 μg each of pcDNA3.1-HisB-cMyc-CaM WT or variants, along with 0.2 μg of the peGFP vector, which codes for the green fluorescence protein as a marker of successful transfection. In certain experiments, 10 nM siRNA targeting the casein kinase 2 α1 (*Csnk2a1*) gene (Ambion, siRNA ID # s138044) or 10 nM of universal negative control siRNA#2 (Sigma-Aldrich, Oakville, ON, Canada) was added. The *Csnk2a1* siRNA target sense and antisense sequences were as follows: *CSNK2A1*, 5′-GGAAGAUUUAUAUGACUtt-3′ and 5′-AUAGUCAUAUAAAUCUUCCgt-3′; negative control, 5′- -3′ and 5′- -3′. This region targets the catalytic domain of the CK2α protein, which encompasses approximately the first 300 amino acids and is essential for the enzyme’s kinase activity. Unfortunately, the Sigma Aldrich company does not divulge the sequence of the universal negative control siRNA#2 (catalog number S1C002-10NMOL).

For experiments involving neonatal cardiomyocytes with only native CaM, the cDNA of pcDNA3.1-HisB-cMyc-CaM WT or variants was not included in the lipofectamine–DNA mix. Transfected cells were initially incubated in a 5% CO_2_ environment at 37 °C for 24 h in a mixture of lipofectamine–DNA solution and culture medium, which was followed by an additional 48 h in fresh DMEM medium before electrophysiological experiments.

When indicated, neonatal cardiomyocytes were treated with either the α-/β1-adrenergic receptor agonist norepinephrine (1 µM) (Sigma-Aldrich, Oakville, ON, Canada) or the β1/β2-adrenergic receptor agonist isoproterenol (100 nM) (Rockville, MD, USA) immediately following transfection. In some other cases, the activity of CK2 was pharmacologically inhibited by directly adding 4,5,6,7-tetrabromobenzotriazole (TBB, 0.5 µM) (Tocris Bioscience, Bristol, UK) to the bath recording solution 15 min prior to the initiation of patch-clamp recordings.

**Western blotting of cultured cardiomyocytes**

Total cell lysates from control and cardiomyocytes transiently transfected with siRNA (see above) were collected 3 days after transfection. Cells were rinsed with PBS 1X and lysed in cold RIPA buffer 1X (catalog #9806; Cell Signaling Technology; 20 mM Tris-HCl (pH 7.5), 150 mM NaCl, 1 mM Na_2_EDTA, 1 mM EGTA, 1% NP-40, 1% sodium deoxycholate, 2.5 mM sodium pyrophosphate, 1 mM beta-glycerophosphate, 1 mM Na_3_VO_4_, 1 µg/mL leupeptin) with 1 mM PMSF (catalog #36978; Thermo Fisher Scientific, Waltham, MA, USA), protease inhibitors (catalog #11836170001, Roche) and phosphatase inhibitors (catalog #P5726 Sigma-Aldrich, Burlington, MA, USA). The lysates were homogenized, briefly sonicated, incubated for 60 min at 4 °C with shaking and centrifuged at 15,000× *g* for 30 min at 4 °C. The supernatant of the protein lysate was collected, and protein concentrations were determined using a BCA assay (catalog #23225; Pierce BCA Protein Assay Kit; Thermo Fisher Scientific). Proteins were mixed with 4x Laemmli and heated for 10 min at 95 °C prior to migration on a 12% SDS-polyacrylamide gel (catalog #4561045, Biorad). The electrophoresed proteins were then transferred onto a nitrocellulose membrane (catalog #1620094, Biorad). The membrane was blocked at room temperature for 30 min using Everyblot blocking buffer (#12010020, Bio-Rad), which was followed by an overnight incubation with CK2α antibody (catalog #2656; 1: 1000, Cell Signaling Technology, Danvers, MA, USA) at 4 °C with agitation. After three 10 min washes with 0.1% TBST, the membrane was incubated for 2 h at room temperature with secondary antibody conjugated with horseradish peroxidase (HRP) and Goat Anti-Rabbit IgG (1:10,000; #111-035-144; Jackson Immuno Research Laboratories, West Grove, PA, USA). After three additional washes with 0.1% TBST, the blot was detected using enhanced chemiluminescence (ECL) (catalog #34075; Supersignal Thermo Fisher) and visualized with the ChemiDoc™ Touch Imaging System (Bio-Rad). Molecular weights were estimated using Precision Plus Protein Dual Color Standards (catalog #1610374, BioRad). After the first detection, the membrane was stripped for 30 min at room temperature with agitation using stripping buffer (catalog #21059, Thermo Fisher Scientific) and reprobed with anti-GAPDH (1: 10,000, catalog #G9545, Sigma-Aldrich) and Anti-Rabbit (1:10,000; catalog #111-035-144; Jackson Immuno Research Laboratories) as loading control, as described above. Molecular weights were estimated using Precision Plus Protein Dual Color Standards (catalog #1610374, BioRad) by linear regression of standard molecular weight markers. The average knockdown in the expression of the CK2 protein was calculated from 5 different cardiomyocyte preparations that were loaded on the same gel as 3 of the same control cardiomyocyte isolations. The signal intensity for each CK2 band was obtained from the ChemiDoc AImage Lab 6.1 software and normalized to the signal intensity of their respective GAPDH loading control. The three fractions were averaged (0.55 ± 0.1) and normalized to 100%. The relative signal intensity for the siRNA bands was also estimated from their respective GAPDH loading controls. The fractional signal of the CK2 protein knockdown was calculated for each well and compared to the averaged control such that

$$\%\;Knockdown=\left(1-\frac{Relative\;signal\;intensity\;CK2\;with\;siRNA}{Mean\;relative\;signal\;intensity\;CK2\;control}\right)\times 100$$

**Patch-clamp experiments**

A whole-cell patch clamp technique was employed to measure L-type Ca^2+^ currents (LTCCs) between 65 h and 75 h post-isolation at room temperature (22~23 °C) using an Axopatch 200B amplifier and pCLAMP 11.2 acquisition software (Molecular Devices, San Jose, CA, USA). Patch electrodes were pulled from borosilicate glass (Corning 8161) with a Narishige PC-10 puller and heat-polished to a final resistance of about 3.5–4.5 MΩ when filled with the intracellular solution. Whole-cell currents were low-pass filtered at 2 kHz, digitized at a sampling rate of 100 µs during acquisition, and stored on a microcomputer equipped with an AD converter (Axon Digidata 1440A, Molecular Devices, Sunnyvale, CA, USA). Cells were bathed in a modified Earle’s saline solution contained (in mM): 135 NMDG, 20 TEACl, 2 CaCl_2_, 1 MgCl_2_, 10 HEPES, titrated to pH 7.4 with HCl. Electrodes were filled with a solution containing (in mM) 140 CsCl, 0.6 NaGTP, 3 MgATP, 10 EGTA, 10 HEPES, which was titrated to a pH of 7.4 with KOH.

All K^+^ currents were blocked with intracellular Cs^+^ and extracellular tetraethylammonium chloride (TEA-Cl). Following a 40 ms prepulse to −40 mV to inactivate the remaining Na^+^ currents and low-voltage activated T-type Ca^2+^ channels, L-type Ca^2+^ currents (LTCCs) were elicited from a holding potential of −80 mV and were depolarized to potentials ranging from −60 to +65 mV in 5 mV increments lasting 450 ms for each step (protocol shown in the inset above the current traces). Inward Ca^2+^ current densities (pA/pF) were obtained by dividing the peak currents by the cell capacitance. Average I–V curves were generated by plotting peak current densities against the applied voltage. The I–V relationships were then fitted to a BoltzIV equation, which is a transformed Boltzmann function tailored for I–V data as the following form:

$$I=\frac{\left(Vm-Vrev\right)\cdot Gmax}{1+e^{(v-E0.5,\;act)/dx}}$$

where *I* is the current, *Vm* is the applied voltage, *E_0.5,act_* is the voltage at which channels are half-maximal activated, *dx* is the steepness of the slope, *Gmax* is the maximal conductance, and *Vrev* is the reversal potential. n/N refers to the number of cells/transfections measured in each condition of study. Only data recorded on day 3 are presented in the figures. The average peak current densities and *E_0.5_,_act_* values on day 2 and 3 are detailed in the table.

**Data analysis and statistics**

Data were analyzed using a combination of pCLAMP software 11.3 (Molecular Devices), Microsoft Excel 2021 (Microsoft, Redmond, WA, USA), and OriginPro 2024 (OriginLab Corporation, Northampton, MA, USA) and expressed as mean ± SD. Statistical significance was determined by Student’s *t*-test, and the level of statistical significance was set at *p* < 0.01.

## 5. Conclusions

Our study revealed that CK2-mediated phosphorylation processes play an unsuspected but essential role in regulating the intrinsic activity of LTCC in cardiomyocytes.

## Figures and Tables

**Table 1:** Biophysical properties of LTCCs from neonatal rat cardiomyocytes.

DNA Transfection		Treatment	Electrophysiological Properties		
CaM	*CSNK2α1* siRNA		n/N	Peak Current Density (pA/pF)	*E_0.5,act_* (mV)
Native CaM	-	-	21/5	−22 ± 6	−10 ± 3
	-	TBB	10/3	−4 ± 2 *	−5 ± 2 *
	-	NE	12/3	−35 ± 5 *	−11 ± 2 **
	-	NE + TBB	15/2	−13 ± 3 *	−6 ± 3 *
	-	ISO	10/2	−33 ± 8 *	−11 ± 1 **
	-	ISO + TBB	5/1	−12 ± 2 *	−8 ± 2
	siRNA	-	10/1	−7 ± 3 *	−4 ± 2 *
	siRNA	NE	7/1	−15 ± 5 *	−7 ± 2
	siRNA	ISO	9/2	−17 ± 3 *	−5 ± 3 *
	siRNA (−)	-	8/1	−25 ± 4	−4 ± 2 *
WT	-	-	16/3	−31 ± 6 *	−8 ± 3
	-	TBB	8/2	−4 ± 1 **	−3 ± 2 **
	-	NE	7/1	−48 ± 8 **	−8 ± 1
	-	NE + TBB	5/1	−14 ± 3 **	−9 ± 2
	siRNA	-	6/1	−6 ± 2 **	−6 ± 2 **
	siRNA	NE	5/1	−19 ± 4 **	−8 ± 2
	siRNA	ISO	10/2	−16 ± 3 **	−6 ± 2
	siRNA (−)	-	9/1	−31 ± 5	−7 ± 2
S81A	-	-	12/2	−9 ± 3 *^,^**	−4 ± 2 *^,^**
	-	TBB	6/1	−6 ± 0.8 ^#^	−3 ± 2
	-	NE	10/2	−20 ± 5 ^#^	−7 ± 2 ^#^
	siRNA	-	11/2	−5 ± 2 ^#^	−2 ± 3
	siRNA	NE	10/3	−19 ± 5 ^#^	−9 ± 3 ^#^
	siRNA	ISO	7/1	−12 ± 3	−9 ± 2 ^#^
	siRNA (-)	-	13/2	−10 ± 3	2 ± 3 ^#^
S81D	-	-	11/3	−37 ± 3 *^,^**	−6 ± 2 *^,^**
	-	TBB	6/1	−3 ± 1 ^#^	−6 ± 1
	siRNA	-	6/1	−5 ± 2 ^#^	−4 ± 2
	-	NE	8/1	−52 ± 6 ^#^	−8 ± 1
	siRNA	NE	10/2	−15 ± 2 ^#^	−11 ± 2 ^#^
	siRNA (−)	-	10/2	−38 ± 5	−3 ± 2 ^#^
T79A	-	-	11/2	−9 ± 4 *^,^**	−2 ± 3 *^,^**
	-	NE	7/1	−20 ± 7 ^#^	−9 ± 2 ^#^
T79D	-	-	9/1	−39 ± 12 *^,^**	−5 ± 2 *^,^**
	siRNA	-	6/1	−5 ± 0.9 ^#^	−4 ± 2
T79A/S81A	-	-	11/1	−9 ± 1 *^,^**	−4 ± 2 *^,^**
T79D/S81D	-	-	7/1	−49 ± 13 *^,^**	−12 ± 2 **
	sRNA	-	10/2	−18 ± 4 ^#^	−8 ± 2 ^#^
T79A/S81A/S101A	-	-	8/1	−6 ± 2 *^,^**	−4 ± 2 *^,^**
	siRNA	-	11/2	−6 ± 2 *^,^**	−3 ± 2 *^,^**
T79D/S81D/S101D	-	-	10/2	−51 ± 5 *^,^**	−11 ± 2 **
	-	TBB	12/2	−23 ± 5 ^#^	−6 ± 2 ^#^
	siRNA	-	14/3	−32 ± 8 ^#^	−6 ± 2 ^#^

The peak current density and *E_0.5,act_* of LTCCs derived from neonatal rat cardiomyocytes were assessed under various conditions: with native CaM, overexpressing CaM WT, phosphoresistant or phosphomimetic variants. Furthermore, the biophysical effects of the CK2 inhibitor TBB, catecholamines (NE and ISO), Csnk2α1 gene silencing via co-transfection of Csnk2α1 siRNA, and co-transfection of universal negative control siRNA on the biophysical properties of LTCCs were evaluated for each condition. The notation n/N represents the number of cells/transfections measured in each experimental condition. Data are presented as mean ± SD. Statistical significance of differences was determined using Student’s *t*-test. Symbols indicate significance levels: *: *p* < 0.01 compared with native CaM; **: *p* < 0.01 compared with CaM WT; ^#^: *p* < 0.01 compared with the corresponding CaM variant.

## Data Availability

Original blots will be made available upon request. All electrophysiological data are reported in the table of the article.
